# Co-selected mutations in *VCP*: a novel mechanism of resistance to VCP inhibitors

**DOI:** 10.1038/s41419-017-0049-9

**Published:** 2018-01-18

**Authors:** Prabhakar Bastola, Jeremy Chien

**Affiliations:** 1 Department of Pharmacology, Toxicology and Therapeutics, University of Kansas Medical Center, Kansas City, Kansas 66160 USA; 2 Division of Molecular Medicine, Department of Internal Medicine, University of New Mexico, Albuquerque, New Mexico 87131 USA

Cells ensure protein homeostasis by maintaining a balance in the rate of protein synthesis and degradation. Dysfunction in protein homeostasis can trigger abnormal cellular functions and result in various disorders. Similarly, cancer cells harbor a host of genomic alterations which can result in the dysregulation of protein expression and the aberrant expression of dysfunctional proteins. Such proteins must be efficiently recycled/degraded through cellular degradation machineries to maintain protein homeostasis. Cancer cells are heavily reliant on protein quality control (PQC) pathways for survival and progression, a process known as the non-oncogenic addiction. Hence, targeting components of the PQC pathways has been shown to be effective as a cancer therapy. Success of proteasome inhibitors such as bortezomib and carfilzomib has provided support to the idea that the ubiquitin-proteasome system, a component of protein homeostasis, could be targeted in cancer.^1,2^ Likewise, effectiveness of compounds such as Hsp90 inhibitors,^3^ autophagy modulators,^4^ and HDAC inhibitors^5^ in several cancer types further support this idea.

VCP/p97, a member of AAA-ATPase family of ATPase, utilizes the hydrolysis of ATP to perform diverse cellular functions.^6^ Over the years, VCP has been implicated with various pathways involved in endoplasmic reticulum associated degradation, proteasome mediated degradation, protein aggregate processing, autophagy, endosomal trafficking, and mitochondria-associated degradation which makes it an important component of protein quality control. The protein has an N-terminal domain that interacts with substrates and cofactors, two ATPase domains (D1 and D2 domains) that bind and hydrolyze ATP; and a short C-terminal region.^7^ Promoted by the evidence of increased VCP expression in cancer^8^ and the emergence of the role of VCP in PQC pathways, several studies have focused in understanding and targeting VCP. CB-5083 is a first-in-class VCP inhibitor that showed favorable pharmacokinetic and pharmacodynamics when administered orally in tumor-bearing mice.^9^ More importantly, CB-5083 showed better efficacy in mouse xenografts solid tumor models in comparison to several proteasome inhibitors. These results allowed for the initiation of two Phase I clinical trials.^9^

Given the therapeutic potential of CB-5083 for targeting solid tumors, we investigated the mechanism of resistance towards this compound in solid tumor. Anderson et al., have previously identified separate homozygous point mutations in the D2 domain as well as D1–D2 linker of VCP protein in CB-5083 resistant cell lines;^9^ however, the extent of overlap in the mechanisms of resistance to CB-5083 and other VCP inhibitors was not explored by the authors. In the recently published paper in Cell Death and Discovery entitled, “Specific mutations in the D1–D2 linker region of VCP/p97 enhance ATPase activity and confer resistance to VCP inhibitors”, we employed a combination of incremental and continuous dosing scheme to establish CB-5083 resistant ovarian cancer cell lines.^10^ We identified two heterozygous mutations, E470D and E470K, in the D1–D2 linker region. This region is close to previously identified point mutations in CB-5083 resistance cell lines,^9^ which further underlies the importance of the D1–D2 linker region in the development of resistance to CB-5083. Furthermore, in vitro VCP ATPase activity assay showed an increase in basal ATPase activity and a higher IC_50_ towards several classes of VCP inhibitors in these VCP-mutant cells compared to parental counterparts. Additionally, we performed unbiased docking to show that E470 is located in a putative CB-5083 binding site. Our results in combination with mutations observed by Anderson et al., suggest D1–D2 linker region as an additional putative binding site for CB-5083.

Besides identifying missense mutation in the D1–D2 linker region, we found heterozygous nonsense mutations at Q603 and N616. Nonsense mutation at N616 is one of the most reported alternations of VCP in all the cancer subtypes, making it a relevant theranostic marker in selecting patients in clinical trials. Furthermore, these nonsense mutations were identified only in genomic DNA sequencing not in the cDNA sequencing of CB-5083 resistant cells. These results suggest nonsense mutations at Q603 and N616 are subjected to nonsense-mediated decay. Consequently, mutant cell lines showed reduced expression of VCP mRNA and protein, which is consistent with the effect of nonsense-mediated decay.

VCP forms hexameric complexes, and our results suggest that inhibition of a wildtype VCP subunit by CB-5083 in a hexameric complex is sufficient for cytotoxicity. Hence, activating mutation in one VCP allele that escapes inhibition by CB-5083 is not sufficient to generate resistance to CB-5083, and the other VCP allele is also subjected to either activating mutation or truncation mutation. In our studies, we observed that the second VCP allele is lost through nonsense mutations (Fig. 1a). Furthermore, previous studies have shown correlation between gene copy number and mRNA level of VCP with resistance to VCP inhibitors,^9^ however the correlation values were low. Our results indicate that mutations in *VCP* should also be taken into consideration when performing such analyzes.

**Fig. 1:**
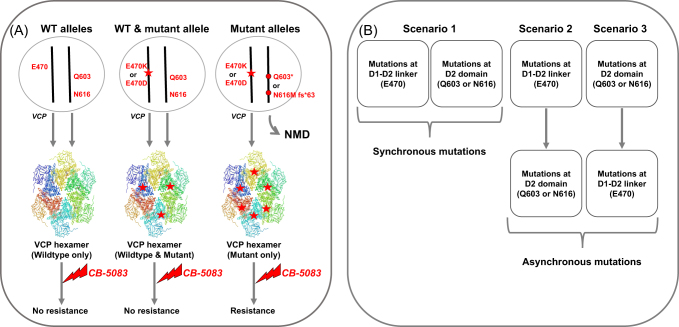
Co-selected mutations in *VCP* confers resistance to CB-5083 . **a** Presence of both wildtype VCP alleles results in wildtype VCP protein hexamer, which can be inhibited by CB-5083. Similarly, presence of one mutant allele (E470K or E470D) result in VCP protein hexamers with mixed VCP proteins (wildtype and mutant). However, inhibition of wildtype protein in the complex upon CB-5083 treatment is sufficient for cytotoxicity. Loss of wildtype copy is required for resistance towards CB-5083. **b** Collective mutations observed upon CB-5083 treatment can occur via three scenarios. Scenario 1 outlines the chance of two different mutations occurring simultaneously; whereas scenario 2 and 3 outline the occurrence of serial mutations whereby mutations at D1–D2 linker is then followed by mutations at D2 domain or vice versa

Our study identified a unique pattern of co-selected mutations with CB-5083 treatment, whereby prolonged treatment allowed for the selection of activating missense mutations at D1–D2 linker region and inactivating nonsense mutations at D2 domain. This co-selection could occur in one of three scenarios as outlined in Fig. 1b. Both mutations may appear synchronously or asynchronously in sequential manner. We observed a progressive establishment of resistance towards CB-5083 which allows us to favor the asynchronous model of resistance, however definitive study needs to be done to confirm this model of resistance. Similarly, further studies are required to identify exact sequence of asynchronous mutations that leads to resistance (scenario 2 or scenario 3). Nonetheless, given that truncation mutation at N616 is found in tumor samples, it is conceivable to suggest that these tumor cells could acquire asynchronous activating mutations in the second allele to become resistant to CB-5083.

Interestingly, although in vitro ATPase assay suggests mutant VCP is cross-resistant to other VCP inhibitors, such as ML240 and DBeQ, CB-5083-resistant cells show comparable sensitivity to ML240 and DBeQ. These results suggest that off-target effects of ML240 and DBeQ contribute to cytotoxicity. It would be important to define additional targets of ML240 and DBeQ, as these cellular targets may be important for further development of therapeutics to overcome resistance to CB-5083.

In conclusion, our study builds upon the idea of target alternation as a potential mode of resistance towards VCP inhibitors. Similarly, we identify novel missense and nonsense mutations that collectively result in resistance to CB-5083. VCP inhibitors treatment induce unfolded protein response (UPR),^11^ which is primarily an adaptive response. CB-5083 could be an effective compound to study the effects of such adaptive responses in the development of resistance in cancer cells. Establishment of resistance cell lines and creation of novel VCP mutant proteins should aid in the development of novel VCP inhibitors as well as provide insight in the understanding of VCP protein which displays such diverse cellular functions.

## References

[1] Chen D (2011). Bortezomib as the First Proteasome Inhibitor Anticancer Drug: Current Status and Future Perspectives. Curr. Cancer Drug Targets.

[2] Kuhn DJ (2007). Blood.

[3] Sidera K (2014). Recent Pat. Anticancer Drug Discov..

[4] Boulay A (2004). Cancer Res..

[5] Santo L (2012). Blood.

[6] Meyer H (2012). Nat. Cell Biol..

[7] DeLaBarre B (2003). Nat. Struct. Biol..

[8] Valle CW (2011). PLoS One.

[9] Anderson DJ (2015). Cancer Cell..

[10] Bastola P (2017). Cell Death Discov..

[11] Bastola P (2016). Mol. Oncol..

